# Pre-Sleep Cognitive Arousal Is Unrelated to Sleep Misperception in Healthy Sleepers When Unexpected Sounds Are Played during Non-Rapid Eye Movement Sleep: A Polysomnography Study

**DOI:** 10.3390/brainsci12091220

**Published:** 2022-09-09

**Authors:** Rachel L. Sharman, Célyne H. Bastien, Michael L. Perlis, Mark A. Wetherell, Nicola L. Barclay, Nayantara Santhi, Jason G. Ellis, Greg J. Elder

**Affiliations:** 1 Sleep and Circadian Neuroscience Institute, Nuffield Department of Clinical Neurosciences, University of Oxford, Oxford OX3 9DU, UK; 2 École de Psychologie, Université Laval, Québec, QC G1V 0A6, Canada; 3 Centre de Recherche CERVO, Québec, QC G1E 1T2, Canada; 4 Behavioral Sleep Medicine Program, Perelman School of Medicine, University of Pennsylvania, 3535 Market Street, Philadelphia, PA 19104, USA; 5 Department of Psychology, Faculty of Health and Life Sciences, Northumbria University, Newcastle upon Tyne NE1 8ST, UK; 6 Sleep Universal Ltd., Oxford OX1 2JD, UK; 7 Northumbria Sleep Research, Department of Psychology, Faculty of Health and Life Sciences, Northumbria University, Newcastle upon Tyne NE1 8ST, UK

**Keywords:** noise, sleep, sleep architecture, sleep misperception, polysomnography

## Abstract

Background: It is well-established that environmental noise can disrupt sleep, and cause a mismatch between subjective and objective sleep, which is known as “sleep misperception”. Naturalistic studies indicate that pre-sleep cognitive arousal and sleep misperception are associated in the context of noise. However, it is not known if this is the case when ecologically valid noises are specifically played during non-rapid eye movement (NREM) sleep, which is susceptible to noise-related disruption. The present study evaluated if pre-sleep cognitive arousal was associated with sleep misperception in healthy normal sleepers, when unexpected ecologically valid common nocturnal noises were played during NREM sleep. Methods: Eighteen healthy sleepers (M_age_ = 23.37 years, SD_age_ = 3.21 years) participated. Sleep was measured objectively on three consecutive nights using polysomnography, in a sleep laboratory environment, and subjectively, through participant estimates of total sleep time (TST). Night 1 was a baseline night where no noises were played. On Night 2, noises, which were chosen to be representative of habitual nocturnal noises heard in home environments, were played to participants via in-ear headphones after 5 min of objective sleep. Results: Unexpectedly, habitual pre-sleep cognitive arousal was not associated with subjective–objective TST discrepancy on Night 2. Conclusions: These results suggest that in healthy sleepers, when ecologically valid noises are played unexpectedly during NREM sleep in an unfamiliar sleep laboratory environment the subjective experience of sleep is not associated with pre-sleep cognitive arousal, or negatively impacted by noise exposure.

## 1. Introduction

Obtaining a sufficient quantity of high-quality sleep has an important role in the maintenance of good physical and psychological health [1]. A loss of sleep, or disruption to sleep, can subsequently lead to a wide range of deleterious cognitive and health outcomes [1,2]. Noise exposure can disrupt both subjective and objective sleep, and this has been consistently observed within both laboratory and naturalistic environments [3,4]. For instance, one laboratory study indicated that relative to a night without noise, auditory noise exposure (road, rail and air traffic sounds) negatively affected objective sleep continuity and sleep architecture [5]. Specifically, these included a reduction in sleep efficiency (SE%), a reduced total sleep time (TST), increased wake after sleep onset (WASO) and an increased latency to slow-wave sleep (SWS) [5], compared to a night where noises were not played. As well as altering objective sleep architecture and continuity, environmental noise can result in objective cortical arousals, expressed as high-frequency electroencephalogram (EEG) activity during sleep, in a dose-response manner [4].

The presence of environmental noise during sleep has the potential to cause poor sleep, and may create or exacerbate the clinical sleep problem of insomnia disorder. Theoretical models of insomnia suggest that cortical arousal, expressed subjectively through increased cognitive activity, and objectively, as increased levels of cortical arousal (e.g., increased non-rapid eye movement (NREM) beta/gamma EEG frequencies), increases sensory and information processing both at sleep onset and during sleep [6,7]. In particular, the neurocognitive model of insomnia suggests that NREM sleep, which is predominantly obtained during the first half of the night [8], is especially vulnerable to disruption by external stimuli such as environmental noise [6]. Indeed, studies show that people with insomnia demonstrate increased levels of cortical arousals, and an association between EEG activity and subjective sleep disturbances [9,10]; this supports the assertion that cortical arousal is involved in the maintenance of insomnia [9,10]. One potential mechanism by which people with insomnia may be particularly vulnerable to sleep disruption is through enhanced sensory processing and memory formation [6,7]. Specifically, increased cortical arousals in insomnia represent not only “shallow sleep”, but also the potential for heightened awareness of external stimuli (enhanced exteroception), which can directly interfere with the perception of sleep. Additionally, memory for such events is thought to negatively influence morning recall/judgement regarding subjective sleep continuity [6,7]. Indeed, electrophysiological evidence suggests that people with insomnia display increased cortical arousal in relation to auditory tones played throughout the night, relative to healthy sleepers [11].

Even healthy sleepers can experience increased sensory processing and sleep disturbances in a noisy environment. This is evidenced by the fact that environmental noise can elicit cortical arousals in healthy sleepers [12]. In experimental situations, whilst healthy sleepers can habituate to environmental noise in terms of subjective sleep, this is not the case for objective sleep, which remains disturbed by noise [13]. Additionally, noise exposure can lead to a direct physiological response by impacting the autonomic system and hypothalamic-pituitary-adrenal (HPA) axis [14,15]. Similarly, despite the habituation effect upon subjective sleep, the cardiac response to noise exposure during sleep does not attenuate during subsequent exposures to noise [16]. Taken together, this indicates that physiologically, healthy sleepers may not be able to fully habituate to noise exposure, and this is relevant as the pathogenesis of insomnia may involve an interaction between increased cognitive and autonomic activation [6,7].

High subjective levels of pre-sleep cognitive arousal, which refers to having high levels of worry about attaining or maintaining sleep, not being able to prevent thoughts occurring, and having a racing mind prior to sleep, are associated with subjective difficulties in falling asleep in adults who do not have insomnia disorder [17,18,19,20,21,22]. Pre-sleep cognitive arousal has also been shown to affect the extent of sleep misperception, which refers to the mismatch between the subjective experience of sleep (e.g., total sleep time (TST) and sleep onset latency, which refer to sleep duration and the time it takes to get to sleep respectively) in both adults with and with-out insomnia disorder [23,24]. Sleep misperception can be quantified by calculating the discrepancy between subjective and objective TST, which is expressed as the percentage of objective sleep (OSE%). An OSE% value of <100% demonstrates that subjectively estimated TST is lower than the objective sleep that is attained; OSE% values of >100% indicate that subjective TST is greater than the objective TST that is attained [25]. Therefore, OSE% values of <100% are indicative of poor perceived sleep quality, and values of >100% of good perceived sleep quality [25].

Habitual pre-sleep cognitive arousal levels can influence the extent to which environ-mental noise disrupts sleep: we have previously demonstrated that in a routine bedroom environment, habitual pre-sleep cognitive arousal is associated with the degree of sleep misperception, which is the difference between the perceived (subjectively measured) total sleep time measured total sleep time (as assessed with polysomnography or actigraphy) [26]. However, in a routine bedroom environment, it is not possible to control for individual variations in bedroom location, and therefore, the timing or duration of environmental noise, which have been shown to differentially affect objective sleep. This is important as intermittent and continuous noise can affect sleep in different ways: for instance, intermittent traffic noise can increase lighter sleep, at the expense of SWS, whereas continuous traffic noise can reduce the amount of rapid eye movement (REM) sleep [27].

The nature of the noise is also likely to be relevant, as whilst is it well-established that specific noises such as wind turbines or traffic noise can disrupt sleep [3,4], much less is known regarding the impact of routine daily household noises, such as voices, pets and footsteps, upon sleep [28]. The environment in which sounds are played may also influence the level of disruption, whereby unfamiliar environments may remove the ability of individuals to habituate. This has been demonstrated in a study which played continuous traffic noise at 50 decibels (dBs) over seven consecutive nights to a group of healthy sleepers who had received the sleep-inducing gamma-aminobutyric agonist Gadoxadol [29]. The traffic noise increased beta EEG activity during the first night of exposure, relative to a baseline night without noise [29]; however, a comparator group, who did not receive Gadoxadol, were unable to habituate and both subjective and objective sleep remained disturbed, even after seven consecutive nights [29].

Taken together, even healthy sleepers might not be able to habituate to noise when the noise disturbance is unexpected, or intermittent, or when they are sleeping in an unfamiliar environment (i.e., an environment which is not a routine home environment). In order to investigate this, a controlled bedroom environment with the objective monitoring of sleep is necessary: this may remove potential protective mechanisms for noise habituation during sleep, and this level of control is not possible in habitual environmental studies [26]. Additionally, the individual trait of sleep reactivity may be relevant, which refers to the degree to which sleep can be disrupted by a challenge, which is typically a broadly defined stressor [30]. Previous work has found that in people with insomnia disorder, sleep reactivity and pre-sleep cognitive arousal are positively associated [31,32]. Finally, memory processing may also be relevant. In a study of healthy sleepers who were presented with auditory stimuli, beta EEG activity was positively associated with the ability to recall played stimuli [33]. However, it is not clear if memory processing of unexpected auditory stimuli is associated with the degree of sleep misperception.

The main aim of the present study was to examine if pre-sleep cognitive arousal was associated with sleep misperception in healthy individuals, when unexpected ecologically valid common nocturnal noises were played during NREM sleep in a sleep laboratory environment. It was hypothesised that, during a night where noises were played during NREM sleep:

(1) Habitual pre-sleep cognitive arousal, but not pre-sleep somatic arousal, would be negatively associated with sleep misperception.
(2) Sleep reactivity would be negatively associated with sleep misperception.
(3) The ability to correctly recall noises on the morning after they were played during NREM sleep, as a marker of memory recall, will be negatively associated with a sleep misperception.

## 2. Materials and Methods

### 2.1. Participants

A total of 18 healthy sleeper participants (M_age_ = 23.37 years, SD_age_ = 3.21 years) were recruited from a staff and student population and from a sleep laboratory recruitment database containing members of the general public. Due to the exploratory nature of the present study, an *a priori* power analysis was not conducted.

Individuals were eligible to participate if they were: (1) aged over 18 and (2) had a stable sleep-wake pattern, which was defined as going to bed and awakening at consistent times, as verified from two weeks of completed subjective sleep diaries and objective sleep monitoring using actigraphy. Individuals were not eligible to participate if they: (1) self-reported a current sleep problem (e.g., insomnia disorder, obstructive sleep apnea, restless legs or periodic limb movement syndrome, etc.), or were taking medication likely to interfere with sleep; (2) were shift workers; (3) reported subjective anxiety or depression (assessed using the Hospital Anxiety and Depression Scale [34]); (4) had a current hearing disorder, or (5) reported trans-meridian travel in the three months prior to participating. All participants provided written informed consent and the study was approved by the Northumbria University Faculty of Health Sciences ethics committee.

### 2.2. Materials and Measures

During the laboratory phase of the study, participants completed the pre-sleep Arousal Scale, to indicate the habitual intensity of their cognitive and somatic arousal prior to sleep (PSAS) [35] and the Ford Insomnia Response to Stress Test (FIRST) [36], as an indicator of the likelihood of experiencing sleeplessness in the context of various stressful situations. The PSAS provides cognitive and somatic arousal subscale scores, which range from 8 to 40; higher scores represent greater habitual levels of pre-sleep arousal [35]. The FIRST is a measure of sleep reactivity, which assesses the extent to which stress exposure can disrupt sleep; scores range from 9 to 36, where higher scores represent greater levels of stress-related vulnerability to sleep disturbances [36].

### 2.3. Noise

Thirty noises were obtained from a specialist audio production company (fonicLAB, Stanmore, UK). These noises were judged by the research team to be ecologically valid and representative of habitual noises heard in UK homes (Table 1). All noises were standardised in duration (3 s) and in their peak frequency (1500 hertz (Hz)). Noises were played at 40 dB, from a desktop PC situated outside the participant bedroom, using a pre-defined playlist (Table 1). The playlist running order was pre-determined on the basis of a previous pilot study, where healthy sleeper volunteers were asked to rate the chosen noises in the order of the expected frequency in their usual home environment (data not shown); consequently, the most frequently reported noises were placed at the beginning of the playlist. Noise stimuli were delivered using in-ear mini-speakers (ear buds) with a frequency range of 20 Hz–22 kHz and a sensitivity of 100 dB (SoundMAGIC, Shoreham-by-Sea, UK). The earphones were secured within the left and the right ear using adhesive surgical tape and were connected to a standard audio extension cable attached to the PC.

**Figure 1:**
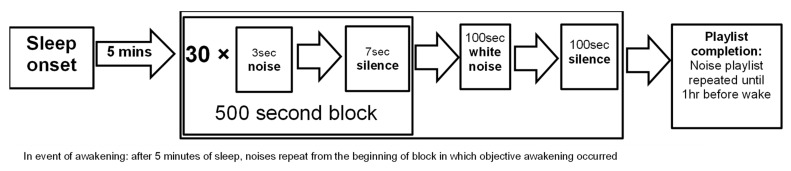
Sleep laboratory noise protocol.

The noise playlist was started five minutes after the onset of objective stage 1 sleep. Noises were played in 500-s blocks, consisting of 30 repetitions of the same noise (played in a pre-determined order; Table 1), where the noise was played for 3 s followed by 7 s of silence. After 30 repetitions, 100 s of white noise was played, which was followed by 100 s of silence. Each subsequent noise was then played in the same way (i.e., 30 × 500 s blocks, before 100 s of white noise and 100 s of silence) until all 30 noises were played. The noise procedure is summarised in Figure 1.

Sleep stage transitions (e.g., from stage 1 to stage 2 of sleep) were ignored. The noise playlist was terminated if participants displayed evidence of awakenings, which was defined as two consecutive 30-s epochs of wake, and restarted from the beginning of the 500-s block after five minutes of objective sleep onset. For example, if a participant awakened half-way through the 500-s block playing Noise 2, then the 500-s block of Noise 2 was restarted when the participant subsequently displayed 5 min of stage 1 sleep. If the noise playlist was completed without objective awakenings being observed, the playlist was repeated until 1 h before the scheduled wake time. All noises were stopped at this time point.

### 2.4. Assessment: Noise Recognition Card Sorting Task

A noise recognition card-sorting task was used to assess the ability of participants to distinguish between auditory noises that were, and were not, presented during the prior night. Participants were presented with a pack of 60 cards, consisting of 30 target noises that were played overnight as part of the noise playlist, and 30 non-target environmental noises which were not played. Participants were instructed to sort the cards into two separate decks of noises which were, and were not, played overnight. Participants were instructed to do this as quickly as possible.

### 2.5. Sleep Measurements

Subjective sleep: Consensus Sleep Diaries (CSD-M) [37] were completed to obtain base-line measures of subjective sleep continuity (TST, time in bed, referring to the period of time between the participant going to bed and leaving bed the subsequent morning (TIB); sleep efficiency (SE%), calculated on the basis of TST/TIB × 100; sleep-onset latency (SOL), referring to the length of time taken to get to sleep; the number of awakenings (NWAK), referring to the frequency of nocturnal awakenings during sleep; and wake after sleep onset (WASO), referring to the duration of time awake during the night after the initiation of sleep.

Objective sleep: Polysomnography (PSG) was applied on each night of the study. EEG electrodes were placed at FP_1_, FP_2_, F_3_, F_4_, C_3_, C_4_, P_3_, P_4_, O_1_, O_2_ and C_z_, referenced to linked mastoids (M_1_, M_2_) and a ground electrode (FP_z_). PSG also included electromyogram (EMG), electrooculogram (EOG) and electrocardiogram (ECG). PSG was obtained using a SOMNOscreen system (SOMNOmedics GmbH, Randersacker, Germany) and EEG impedance levels were maintained below 5 kΩ.

The noise exposure protocol was initiated on the basis of automatic sleep scoring con-ducted using the in-built PSG data acquisition software (DOMINO v1.3; SOMNOmedics GmbH, Randersacker, Germany). PSG recordings were blind scored in 30-s epochs by an external scorer in accordance with standard criteria [38]. From objective PSG data, measures of sleep continuity (TST; SE%; SOL; NWAK; WASO) and sleep architecture (the percentages of wake, and percentages of sleep spent in rapid eye movement (REM), stage 1, stage 2, stage 3 and stage 4 sleep, and the latency to each stage of sleep) were derived.

### 2.6. Procedure

**Figure 2:**
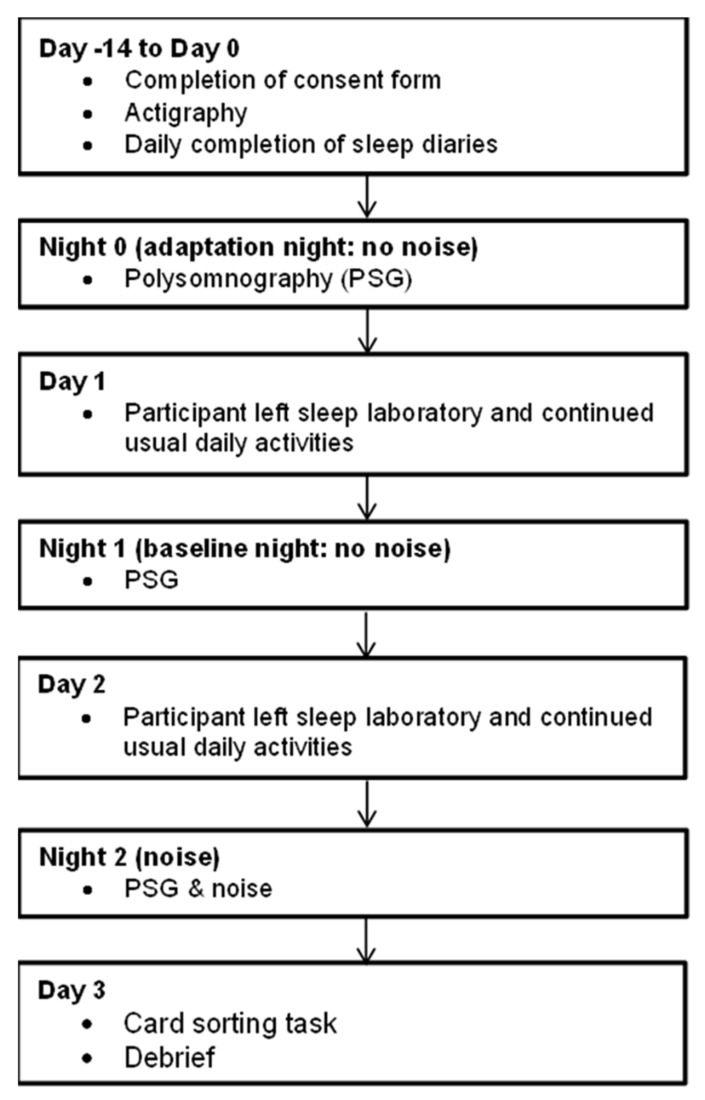
Study procedure (PSG: polysomnography).

The study procedure is summarised in Figure 2. Participants initially attended the sleep laboratory to provide informed consent and be confirmed as a good sleeper by a member of the research team, by examining their sleep, psychiatric and physical illness history.

The study consisted of a baseline period (Day −14 to Day 0) and a three-night weekday sleep laboratory period (Night 0–Night 2). During the baseline period (Day −14 to Day 0), participants completed self-report sleep diaries [37], and continuously wore accelerometers on the non-dominant wrist (Actiwatch AW4, Cambridge Neurotechnologies, Cambridge, UK), to obtain habitual sleep/wake information. During the sleep laboratory phase (Night 0 to Night 2), participants slept for three consecutive weekday nights in a noise-attenuated sleep laboratory bedroom, where sleep was measured objectively using PSG. Participants wore in-ear speakers on all three nights.

Upon arrival at the sleep laboratory on Night 0, sleep diaries and actigraphy information were examined to verify the stability of participant sleep/wake schedules. Sleep and wake times were then scheduled in accordance with average baseline (Day −14 to Day 0) weekday sleep/wake times, as derived from baseline sleep diaries. Participants were not permitted to take mobile phones or other electronic devices into the bedrooms on any night.

Participants were asked to estimate their subjective TST on each morning. On Day 1 and Day 2, participants left the sleep laboratory approximately 90 min after completion of the sleep diary. Participants were instructed to resume their routine daily activities, but to avoid napping and avoid consuming alcoholic drinks, or caffeinated drinks after 6 pm, before returning to the sleep laboratory on Night 1 and Night 2.

Night 1 was considered to be a baseline night, and noises were not played. Noises were only played on Night 2, as firstly, this fixed schedule eliminated the risk of noise having a residual effect upon sleep during the following night; secondly, this ensured that participants did not anticipate noises being played on every night in the sleep laboratory, as even anticipating a nocturnal noise disturbance can disturb sleep in a laboratory environment [39]. On Day 3, participants completed the noise recognition card sorting task, before completing a brief hearing test, where they were asked to verbally confirm that they heard a 1000 Hz tone, played at 40 dB. Participants were then debriefed and received a payment of 150 GBP for their time.

### 2.7. Data Analysis

PSG data from Night 0 were not analysed as objective sleep is disrupted during the first night in a sleep laboratory [40]. PSG data from four participants were excluded from objective sleep continuity and architecture analyses; this was due to an inability to attain stage 4 sleep on either Night 1 or 3 (*n* = 3) or REM sleep on Night 2 (*n* = 1). One participant was unable to provide an estimate of subjective TST and was excluded on this basis.

The subjective-objective sleep discrepancy (i.e., sleep misperception) was obtained by measuring the difference between sleep diary and PSG-measured TST. This was performed by calculating the percentage of objective sleep estimated (OSE%) = ((TST_subjective_/TST_PSG_) × 100) for Night 1 and Night 2. A percentage value of <100% indicates that the subjective estimate of TST is lower than the actual obtained TST; a value of 100% indicates perfect concordance between subjective and objective TST, and a percentage value of >100% indicates that the subjective TST is greater than the obtained TST [25].

To examine the association between habitual pre-sleep cognitive and somatic arousal, and sleep misperception, Pearson correlations were conducted between PSAS cognitive and somatic scores and OSE% values, with *p*-values adjusted for multiple comparisons (corrected *p*-value = 0.025). Associations between sleep reactivity and sleep misperception were examined by conducting Pearson correlations between FIRST and Night 2 OSE% values. In order to examine if noise recall performance was associated with sleep misperception, Pearson correlations were conducted between the percentage of correctly identified sounds on Day 3 and Night 2 OSE% values. Finally, to examine the association between objective wake and noise recall. Effect sizes are reported using *d*_z_, *η*^2^_p_ or *r*, where appropriate. Due to the small sample size, these analyses were repeated using non-parametric tests; however, as the overall results were the same, the results of parametric statistical analyses are reported.

### 2.8. Additional Analyses

Additional analyses examined whether objective sleep differed between Night 1 and Night 2. Three separate repeated-measures multivariate analyses of variance (MANOVAs) were used to compare objective sleep continuity (SOL, TST, NWAK and SE%) and objective sleep architecture (percentages of wake, Stage 1, Stage 2, Stage 3, Stage 4 and REM) and the latencies to each stage of objective sleep (Stage 1, Stage 2, Stage 3, Stage 4 and REM). Due to the small sample size, these analyses were repeated using non-parametric tests (non-parametric MANOVAs were conducted using the *npmv* package (version 2.4.0) for R [41]: https://cran.r-project.org/web/packages/npmv/index.html (accessed on 8 September 2022); however, as the overall results were the same, the results of parametric statistical analyses are reported.

Finally, to explore if high and low and habitual pre-sleep cognitive activity might influence OSE% values, participants were divided into those with and without potentially clinically significant levels of pre-sleep cognitive arousal. In line with previous work demonstrating that a PSAS cognitive subscore of 20 is an appropriate cut-off score for clinically significant pre-sleep activity [22], participants with a PSAS cognitive subscore of ≥20 were considered to have high presleep cognitive activity (*n* = 11, *M* = 21.73), and those with a score of <20 were considered to have low presleep cognitive activity (*n* = 7, *M* = 13.71). OSE% scores were compared between high and low pre-sleep cognitive arousal groups using a non-parametric Mann–Whitney *U* test.

## 3. Results

A total of 17 participants provided subjective TST data (M_age_ = 22.96 years; SD_age_ = 2.79 years) and 14 participants provided complete objective sleep data (M_age_ = 22.98 years; SD_age_ = 2.94 years). Demographic and relevant questionnaire results are shown in Table 2. Summary subjective and objective sleep is presented in Table 3 and Table 4.

Overall, the association between pre-sleep cognitive arousal and Night 2 OSE% values was not significant (PSAS cognitive: *r* = 0.23 and PSAS somatic: *r* = 0.13, *p*-values > 0.025). FIRST scores, as a measure of sleep reactivity, were not significantly related to Night 2 OSE% values (*r* = 0.41, *p* > 0.05), There was no association between Night 2 OSE% values and Day 3 noise recall performance (*r* = 0.14, *p* > 0.05).

Noise presentation during sleep did not negatively affect subjective TST, as there were no significant differences in subjective TST between Night 1 and Night 2 (*t*(16) = 1.74, *p* > 0.05, *d*_z_ = 0.42). The presentation of noise had no impact on usual objective sleep parameters of sleep continuity (*F*(4, 23) = 1.87, *p* > 0.05, *η*^2^_p_ = 0.25) objective sleep architecture (*F*(6, 21) = 1.97; *p* > 0.05, *η*^2^_p_ = 0.36) or the latency to any stage of objective sleep (*F*(5, 22) = 0.94, *p* > 0.05; *η*^2^_p_ = 0.18), relative to a night without noise (Table 4).

There was no significant difference between high and low pre-sleep cognitive arousal groups in terms of Night 2 OSE% values (*U* = 16.00, *z* = −31, *p* > 0.05).

## 4. Discussion

The primary aim of this study was to examine if pre-sleep cognitive arousal was associated with sleep misperception in healthy individuals, when unexpected, common and ecologically valid nocturnal noises were played during NREM sleep in an unfamiliar sleep laboratory environment. The main results of the study indicated that unexpectedly, neither pre-sleep cognitive or somatic arousal was associated with sleep misperception during Night 2, when nocturnal noises were played. Furthermore, neither the degree of habitual sleep reactivity, nor noise recall ability during the morning after noises were played, were associated with Night 2 sleep misperception.

These results indicate that overall, when ecologically valid noises are played unexpectedly during NREM sleep in an unfamiliar sleep laboratory environment, habitual pre-sleep cognitive arousal levels are not associated with the resulting degree of sleep misperception. This indicates that in healthy sleepers, the subjective experience of sleep is not negatively impacted by ecologically valid noise exposure during NREM sleep. Although in experimental studies, healthy sleepers have demonstrated cortical arousals, and have been shown to be unable to habituate to noise (aircraft noises with a maximum sound pressure level of 65 dB (A)), in terms of objective sleep [12,13], this suggests that healthy sleepers can quickly habituate to ecologically valid environmental noises. This appears to be the case even within a non-routine environment; speculatively, healthy sleepers may have a protective ‘buffer’ against noises below a certain threshold. The subjectively low levels of Night 2 noise recall (approx. 25%) would appear to support this notion, a similar study involving people with insomnia observed recall rates of approximately 70% [42]. Clinically, this suggests that healthy sleepers may have a protective buffer which prevents noise exposure from subsequently negatively affecting subjective sleep, which maintains good perceived sleep quality and may prevent the transition to poor sleep. Although these results are not in line with previous work that has observed a relationship between pre-sleep cognitive arousal and sleep misperception [24,26], these previous studies were conducted in a routine habitual environment, and not a sleep laboratory. This would indicate that experimentally induced unusual noises affect sleep more when they are played in a habitual environment, as compared to a novel environment; speculatively, this could be due to individuals being more inclined to actively monitor their habitual environment for unusual sounds (as, for instance, unusual sounds may be perceived as being threatening); in a non-routine environment, a healthy sleeper may not necessarily perceive unusual noises as being relevant or threatening. Additionally, as the nature of the noises employed in previous studies differ: wind turbines, or traffic noise can disrupt sleep [3,4,29], our results also indicate that the *nature* of the noise is relevant.

There are several reasons for the unexpected findings of the present study. The primary reason is likely to be the noises chosen in the present study were not disturbing, or perturbing, enough to disrupt sleep. Whilst the noises were considered to be representative of habitual environmental noise, and included noises such as household noises (e.g., telephones ringing, toilets flushing) or traffic noises, these may not have been personally relevant: it has been shown that personally relevant, or threatening sounds, may have been more disruptive to sleep [43]. To the best of our knowledge, the present study is one of the first to specifically examine the impact of routine environmental noise upon sleep misperception, and polysomnographically measured objective sleep, in a controlled sleep laboratory environment.

Secondly, the noise exposure in the present study specifically targeted NREM sleep, due to the vulnerability to disruption caused by external stimuli [6,8]. However, despite the strong theoretical basis for targeting NREM in the present study, lower overall awakening thresholds are observed in REM sleep relative to NREM [44], and noise exposure may actually have more of an impact during this stage; future studies should specifically examine if this is the case. Thirdly, it is possible that the noises presented may not have been loud enough to impact upon sleep. Although in the present study, noises were played at 40 dB, which is in line with previous noise exposure studies, and above others [4,43], it is possible that the noises may have needed to have been adjusted to individual hearing levels to be effective. This was mitigated against to an extent, as participants were required to verify that they had heard the noise prior to sleep; however, future studies should specifically examine this.

The present study could be extended by examining specific individual differences that may have influenced the impact of noise upon sleep, since previous work has demonstrated that individual differences can explain the degree of noise-induced sleep disturbances [13]. Whilst in the present study, we observed that there was no difference in sleep misperception between individuals with low or high pre-sleep cognitive arousal levels, defined on the basis of a clinically significant cut-off value [22], it should be noted that this was exploratory and studies with sufficient statistical power should examine this further. Additionally, other stress vulnerability may have an impact [36]. Whilst it was beyond the scope of the present study to specifically categorise individuals on the basis of the risk of their stress-related vulnerability to sleep disturbances [36], or low/high habitual cognitive or somatic arousal levels [45], it could be examined if this has an impact upon sleep misperception in the context of noise.

Whilst this was also beyond the scope of the present study, specific features of objective sleep are worthy of further investigation, given their potential protective role. Specifically, K-complexes, which are distinctive, features of NREM sleep, consisting of a negative slow wave followed by a positive slow wave of more than 0.5 s in duration [46], and sleep spindles, which are brief oscillatory events (i.e., ‘bursts’ of brain activity), in the sigma range of approximately 11–15 Hz, which last between 0.5 and 3 s [47] are of relevance. When a K-complex is evoked using a stimulus, rather than occurring spontaneously, they are thought to function as a protective mechanism in healthy sleepers, whereby they prevent NREM sleep from being disrupted [46,48]. Similarly, sleep spindles, which relay sensory information from the thalamus to cortical regions via a thalamocortical feedback loop [47,49,50,51], may have a role in gating sensory information during sleep: it has been shown that spindles act as a sleep stability mechanism, ensuring that sleep continues uninterrupted in a noisy sleep environment [52]: elevated K-complex and/or sleep spindle activity may have afforded healthy sleepers a certain amount of protection against noises. Finally, it should be noted that study participants were healthy young adults, with a mean age of approximately 23 years. It has been shown that with increasing age, awakenings in response to auditory tones are more likely to occur [44]; as such, our participants may be less susceptible to sleep disruption induced by environmental noise compared to older adults.

The present study had several strengths. Firstly, the highly controlled sleep laboratory environment ensured that noises were played in a consistent manner, and that we were able to objectively verify that participants were in NREM sleep, ensuring targeted disruption. Whilst previous environmental studies do demonstrate that noises are disruptive to objective sleep [3,4], they are unable to target specific stages of sleep. Additionally, we ensured that participants were healthy sleepers with a stable sleep-wake cycle. Finally, we conducted pilot testing of the noises before proceeding with the sleep laboratory study to ensure that the noises were representative of environmental noises heard in habitual bedroom environments. Despite the strengths of the study, there are two main potential methodological limitations. Firstly, it is possible that by requiring participants to wear in-ear speakers during each night of the sleep laboratory phase, which were used to ensure that they were exposed to noises at a consistent level, the participants may have correctly predicted that they would have been exposed to noise on the final night, given that no noise was presented on prior occasions. This may have had a potential additional protective effect upon objective sleep; however, this was necessary to ensure that participants were comfortable sleeping using in-ear speakers, and to provide baseline objective sleep data for comparison. Future studies should examine whether this has a role in the level of disruption. Secondly, whilst the choices of noises were piloted to ensure that they were representative of usual habitual environments, we could not ensure that these were personally relevant at the participant level; as discussed, this may have had an impact upon sleep misperception [43]. The present study also had two further potential minor methodological limitations which could be addressed in future studies. Firstly, the relatively small sample size meant that we were unable to examine potential individual differences in factors such as hearing levels. Secondly, the use of participant-reported questionnaires can potentially lead to response bias [53]; therefore, future studies may wish to assess other measures of pre-sleep arousal.

## 5. Conclusions

Overall, the results of the present study indicated that pre-sleep cognitive arousal was not associated with sleep misperception in healthy individuals, when unexpected common nocturnal noises were played during NREM sleep in an unfamiliar sleep laboratory environment.

## Figures and Tables

**Table 1:** Noise stimuli administered to participants (listed in playlist order).

Order	Noise
1.	Wind on the Windows
2.	Clock ticking
3.	House Creaking
4.	Toilet Flushing
5.	Phone Ringing
6.	Rain on the Window
7.	Tap Dripping
8.	Cats Fighting
9.	Car Alarm
10.	People Arguing
11.	Cat Meowing
12.	Keys Rattling
13.	Police Car Siren
14.	Rain Falling
15.	Music from Neighbours
16.	Traffic
17.	Fridge Humming
18.	Fire Engine Siren
19.	Dog Barking
20.	Ambulance Siren
21.	Fireworks
22.	Birds Chirping
23.	Hail Stones falling
24.	Wind in the Trees
25.	Washing Machine
26.	Thunderclap
27.	Gravel Being Blown
28.	TV Static
29.	Litter Being Blown
30.	Milk Float

**Table 2:** Participant demographics (*n* = 17).

	Mean	SD
Age	22.96	2.79
Gender (male/female; *n* (%))	9 (52.9%)/8 (47.1%)	
PSAS (cognitive)	18.94	4.21
PSAS (somatic)	10.35	3.18
FIRST	17.76	3.36
Card sorting task: correctly identified sounds (%)	24.32	18.06

Abbreviations: PSAS: Presleep Arousal Scale; FIRST: Ford Insomnia Response to Stress Test.

**Table 3:** Summary of subjective sleep from sleep laboratory study phase (*n* = 17).

	Night 1		Night 2	
	Mean	SD	Mean	SD
Subjective TST (min)	437.65	35.58	418.82	50.98
OSE%	102.90	6.02	105.00	17.56

Abbreviations: OSE%: percentage of objective sleep estimated; TST: total sleep time.

**Table 4:** Objective sleep continuity and architecture comparisons between noise and no noise nights (*n* = 14).

	Night 1 (No Noise)		Night 2 (Noise)	
	Mean	SD	Mean	SD
TST (min)	427.91	26.88	409.57	37.85
SOL (min)	20.21	15.60	15.38	9.90
NWAK	5.43	5.64	7.93	3.85
WASO (min)	22.57	14.52	42.00	28.37
SE (%)	90.86	4.11	87.79	5.98
Time in REM (%)	22.35	6.69	19.33	6.07
Time in Stage 1 (%)	4.16	2.14	5.42	1.82
Time in Stage 2 (%)	54.83	6.05	56.87	6.00
Time in Stage 3 (%)	13.65	3.68	13.01	2.55
Time in Stage 4 (%)	5.01	3.80	5.38	4.15
Latency to REM (min)	87.88	35.79	116.76	56.29
Latency to Stage 2 (min)	22.30	15.48	18.94	10.80
Latency to Stage 3 (min)	35.11	16.17	37.83	31.89
Latency to Stage 4 (min)	43.47	16.99	56.37	52.43

Abbreviations: TST: total sleep time, SOL: sleep onset latency, NWAK: number of awakenings, WASO: wake after sleep onset, SE: sleep efficiency, REM: rapid eye movement sleep.

## Data Availability

The data presented in this study are available upon reasonable re-quest from the corresponding author. The data are not publicly available due to privacy reasons.

## References

[1] Buysse D.J. (2014). Sleep health: Can we define it? Does it matter?. Sleep.

[2] Grandner M.A. (2020). Sleep, Health, and Society. Sleep Med. Clin..

[3] Hume K.I., Brink M., Basner M. (2012). Effects of environmental noise on sleep. Noise Health.

[4] Basner M., McGuire S. (2018). WHO Environmental Noise Guidelines for the European Region: A Systematic Review on Environmental Noise and Effects on Sleep. Int. J. Environ. Res. Public. Health.

[5] Griefahn B., Marks A., Robens S. (2006). Noise emitted from road, rail and air traffic and their effects on sleep. J. Sound Vib..

[6] Perlis M.L., Giles D.E., Mendelson W.B., Bootzin R.R., Wyatt J.K. (1997). Psychophysiological insomnia: The behavioural model and a neurocognitive perspective. J. Sleep Res..

[7] Riemann D., Nissen C., Palagini L., Otte A., Perlis M.L., Spiegelhalder K. (2015). The neurobiology, investigation, and treatment of chronic insomnia. Lancet Neurol..

[8] Medic G., Wille M., Hemels M.E. (2017). Short- and long-term health consequences of sleep disruption. Nat. Sci. Sleep.

[9] Krystal A.D., Edinger J.D., Wohlgemuth W.K., Marsh G.R. (2002). NREM sleep EEG frequency spectral correlates of sleep complaints in primary insomnia subtypes. Sleep.

[10] Perlis M.L., Smith M.T., Andrews P.J., Orff H., Giles D.E. (2001). Beta/Gamma EEG activity in patients with primary and secondary insomnia and good sleeper controls. Sleep.

[11] Bastien C.H., St-Jean G., Morin C.M., Turcotte I., Carrier J. (2008). Chronic psychophysiological insomnia: Hyperarousal and/or inhibition deficits? An ERPs investigation. Sleep.

[12] Frase L., Maier J.G., Zittel S., Freyer T., Riemann D., Normann C., Feige B., Nitsche M.A., Nissen C. (2015). Bifrontal Anodal Transcranial Direct Current Stimulation (tDCS) Improves Daytime Vigilance and Sleepiness in a Patient With Organic Hypersomnia Following Reanimation. Brain Stimul..

[13] McGuire S., Muller U., Elmenhorst E.M., Basner M. (2016). Interindividual Differences in the Effects of Aircraft Noise on Sleep Fragmentation. Sleep.

[14] Munzel T., Schmidt F.P., Steven S., Herzog J., Daiber A., Sorensen M. (2018). Environmental Noise and the Cardiovascular System. J. Am. Coll. Cardiol..

[15] Waye K.P., Clow A., Edwards S., Hucklebridge F., Rylander R. (2003). Effects of nighttime low frequency noise on the cortisol response to awakening and subjective sleep quality. Life Sci..

[16] Griefahn B., Brode P., Marks A., Basner M. (2008). Autonomic arousals related to traffic noise during sleep. Sleep.

[17] Alapin I., Libman E., Bailes S., Fichten C.S. (2003). Role of nocturnal cognitive arousal in the complaint of insomnia among older adults. Behav. Sleep. Med..

[18] Fichten C.S., Libman E., Creti L., Amsel R., Sabourin S., Brender W., Bailes S. (2001). Role of Thoughts During Nocturnal Awake Times in the Insomnia Experience of Older Adults. Cogn. Ther. Res..

[19] Yeh Z.-T., Wung S.-K., Lin C.-M. (2015). Pre-sleep arousal as a mediator of relationships among worry, rumination, and sleep quality. Int. J. Cogn. Ther..

[20] Ruivo Marques D., Allen Gomes A., Nicassio P.M., Azevedo M.H.P. (2018). Pre-Sleep Arousal Scale (PSAS): Psychometric study of a European Portuguese version. Sleep Med..

[21] Jansson-Frojmark M., Norell-Clarke A. (2012). Psychometric properties of the Pre-Sleep Arousal Scale in a large community sample. J. Psychosom. Res..

[22] Puzino K., Amatrudo G., Sullivan A., Vgontzas A.N., Fernandez-Mendoza J. (2020). Clinical Significance and Cut-Off Scores for the Pre-Sleep Arousal Scale in Chronic Insomnia Disorder: A Replication in a Clinical Sample. Behav. Sleep. Med..

[23] Herbert V., Pratt D., Emsley R., Kyle S.D. (2017). Predictors of Nightly Subjective-Objective Sleep Discrepancy in Poor Sleepers over a Seven-Day Period. Brain Sci..

[24] Takano K., Boddez Y., Raes F. (2016). I sleep with my Mind’s eye open: Cognitive arousal and overgeneralization underpin the misperception of sleep. J. Behav. Ther. Exp. Psychiatry.

[25] Edinger J.D., Fins A.I. (1995). The distribution and clinical significance of sleep time misperceptions among insomniacs. Sleep.

[26] Sharman R.L., Perlis M.L., Bastien C.H., Barclay N.L., Ellis J.G., Elder G.J. (2022). Pre-Sleep Cognitive Arousal Is Negatively Associated with Sleep Misperception in Healthy Sleepers during Habitual Environmental Noise Exposure: An Actigraphy Study. Clocks Sleep.

[27] Eberhardt J.L., Stråle L.O., Berlin M.H. (1987). The influence of continuous and intermittent traffic noise on sleep. J. Sound Vib..

[28] Omlin S., Bauer G.F., Brink M. (2011). Effects of noise from non-traffic-related ambient sources on sleep: Review of the literature of 1990–2010. Noise Health.

[29] Dijk D.J., Stanley N., Lundahl J., Groeger J.A., Legters A., Trap Huusom A.K., Deacon S. (2012). Enhanced slow wave sleep and improved sleep maintenance after gaboxadol administration during seven nights of exposure to a traffic noise model of transient insomnia. J. Psychopharmacol..

[30] Kalmbach D.A., Anderson J.R., Drake C.L. (2018). The impact of stress on sleep: Pathogenic sleep reactivity as a vulnerability to insomnia and circadian disorders. J. Sleep Res..

[31] Palagini L., Mauri M., Dell’Osso L., Riemann D., Drake C.L. (2016). Trait- and pre-sleep-state-dependent arousal in insomnia disorders: What role may sleep reactivity and sleep-related metacognitions play? A pilot study. Sleep Med..

[32] Walker J.L., Vargas I., Drake C.L., Ellis J.G., Muench A., Perlis M.L. (2022). The Natural History of Insomnia: High Sleep Reactivity Interacts with Greater Life Stress to Predict the Onset of Acute Insomnia. Sleep.

[33] Wyatt J.K., Bootzin R.R., Allen J.J., Anthony J.L. (1997). Mesograde amnesia during the sleep onset transition: Replication and electrophysiological correlates. Sleep.

[34] Zigmond A.S., Snaith R.P. (1983). The Hospital Anxiety and Depression Scale. Acta Psychiatr. Scand..

[35] Nicassio P.M., Mendlowitz D.R., Fussell J.J., Petras L. (1985). The phenomenology of the pre-sleep state: The development of the pre-sleep arousal scale. Behav. Res. Ther..

[36] Drake C.L., Richardson G., Roehrs T., Scofield H., Roth T. (2004). Vulnerability to stress-related sleep disturbance and hyperarousal. Sleep.

[37] Carney C.E., Buysse D.J., Ancoli-Israel S., Edinger J.D., Krystal A.D., Lichstein K.L., Morin C.M. (2012). The consensus sleep diary: Standardizing prospective sleep self-monitoring. Sleep.

[38] Rechtschaffen A., Kales A. (1968). A Manual of Standardized Terminology, Techniques and Scoring System for Sleep Stages of Human Subjects.

[39] Wuyts J., De Valck E., Vandekerckhove M., Pattyn N., Exadaktylos V., Haex B., Maes J., Verbraecken J., Cluydts R. (2012). Effects of pre-sleep simulated on-call instructions on subsequent sleep. Biol. Psychol..

[40] Toussaint M., Luthringer R., Schaltenbrand N., Carelli G., Lainey E., Jacqmin A., Muzet A., Macher J.P. (1995). First-night effect in normal subjects and psychiatric inpatients. Sleep.

[41] R Development Core Team (2021). R: A Language and Environment for Statistical Computing.

[42] Perlis M.L., Smith M.T., Orff H.J., Andrews P.J., Giles D.E. (2001). The mesograde amnesia of sleep may be attenuated in subjects with primary insomnia. Physiol. Behav..

[43] Muzet A. (2007). Environmental noise, sleep and health. Sleep Med. Rev..

[44] Busby K.A., Mercier L., Pivik R.T. (1994). Ontogenetic variations in auditory arousal thresh-old during sleep. Psychophysiology.

[45] Puzino K., Frye S.S., LaGrotte C.A., Vgontzas A.N., Fernandez-Mendoza J. (2019). Am I (hyper)aroused or anxious? Clinical significance of pre-sleep somatic arousal in young adults. J. Sleep Res..

[46] Forget D., Morin C.M., Bastien C.H. (2011). The role of the spontaneous and evoked k-complex in good-sleeper controls and in individuals with insomnia. Sleep.

[47] Luthi A. (2014). Sleep Spindles: Where They Come From, What They Do. Neuroscientist.

[48] Halasz P. (2005). K-complex, a reactive EEG graphoelement of NREM sleep: An old chap in a new garment. Sleep Med. Rev..

[49] Baran B., Karahanoğlu F.I., Mylonas D., Demanuele C., Vangel M., Stickgold R., Anticevic A., Manoach D.S. (2019). Increased thalamocortical connectivity in schizophrenia correlates with sleep spindle deficits: Evidence for a common pathophysiology. Biol. Psychiatry Cogn. Neurosci. Neuroimaging.

[50] Steriade M. (2006). Grouping of brain rhythms in corticothalamic systems. Neuroscience.

[51] Astori S., Wimmer R.D., Lüthi A. (2013). Manipulating sleep spindles—Expanding views on sleep, memory, and disease. Trends Neurosci..

[52] Dang-Vu T.T., McKinney S.M., Buxton O.M., Solet J.M., Ellenbogen J.M. (2010). Spontaneous brain rhythms predict sleep stability in the face of noise. Curr. Biol..

[53] Althubaiti A. (2016). Information bias in health research: Definition, pitfalls, and adjustment methods. J. Multidiscip. Healthc..

